# Notes on *Ipomoea* L. (Convolvulaceae) in Cuba and neighbouring islands with a checklist of species found in Cuba

**DOI:** 10.1007/s12225-017-9717-2

**Published:** 2017-09-21

**Authors:** J. R. I. Wood, R. W. Scotland

**Affiliations:** 1 Department of Plant Sciences, University of Oxford, South Parks Road, Oxford, OX1 3RB UK; 2 Honorary Research Associate, Royal Botanic Gardens, Kew, Richmond, Surrey, TW5 3AB UK

**Keywords:** Brazil, Caribbean, endemism, Hispaniola, Jamaica, lectotypes, taxonomy

## Abstract

An updated checklist of species of *Ipomoea* L. found in Cuba is presented with analysis of the different elements represented. *I. alterniflora* Griseb. is defined broadly to include *I. obtusata* Griseb. and *I. excisa* Urb. and its differences from the little-known *I. cubensis* (House) Urb. are discussed. *I. calophylla* C. Wright ex Griseb. is reinstated as the correct name for the species generally known as *I. lacteola* House. *I. praecox* C. Wright is recognised as a distinct species from *I. argentifolia* A. Rich. and images are provided to help distinguish the two species. *I. flavopurpurea* Urb. and *I. dajabonensis* Alain are shown to be conspecific with *I. longeramosa* Choisy, whose disjunct distribution is mapped and discussed. The little-known *I. montecristina* Hadač is described and illustrated and the cited collections show it to be locally common in the Guantánamo region. *I. microdonta* J. R. I. Wood & Scotland is described as new from Camagüey in central Cuba. Eight species endemic to Cuba collected by Ekman and described by Urban in 1924 – 25 are evaluated but only two, *I. balioclada* Urb. and *I. erosa* Urb., are deemed to warrant recognition as distinct endemic species. The origin and typification of *I. horsfalliae* Hook. are discussed and an epitype designated. Cultivated plants named *I. horsfalliae* occur in many tropical countries including Cuba but their extreme variation suggests hybrid origin. Four species from Jamaica, *I. rubella* House, *I. lineolata* Urb., *I. carmesina* Proctor and the Jamaican plant called *I. horsfalliae* are treated as synonyms of a variable *I. lineolata*, which is endemic to the island. *I. saxicola* Proctor is treated as var. *saxicola* J. R. I. Wood & Scotland of *I. ternata* Jacq. *I. cyanantha* Griseb. is treated as a synonym of *I. lindenii* M. Martens & Galeotti. Lectotypes are designated for *I. cyanantha, I. lindenii, I. praecox*, *I. punctata* C. Wright, *I. geranioides* Meisn. and *I. grisebachii* Urb.

## Introduction

There are approximately 430 species of *Ipomoea* found in the New World, an estimate based on the list published by Austin & Huáman (1996), on various more recent publications and on our own on-going research as part of the *Ipomoea* “Foundation Monograph” project (Wood *et al.* 2015, 2017; Wood & Scotland 2017a, 2017b). As a generalisation it can be stated that the genus is more or less restricted to regions with a tropical or subtropical climate and very few species occur at higher latitudes than 30°. However, species diversity is far from uniform. Few species grow above 2000 m and there are remarkably few in the more humid lowland forest areas, particularly the Amazon basin (Wood & Scotland 2017b). The lack of diversity in Colombia appears genuine and is remarkable given the variety of habitats and climates in that country. Diversity is particularly great around the 20 – 25° latitudes in Mexico and in the southern hemisphere in southern Bolivia, northern Argentina, Paraguay and south-central Brazil (Wood & Scotland 2017a, b).

Another interesting feature is the presence of around 20 – 25 very widespread species in nearly every tropical country both in the Old and New Worlds. These species constitute 50% or more of the *Ipomoea* flora in countries where the genus is less diverse but usually amount to around 20% of the total even in the countries with the most diverse floras. These very widespread species are often of imprecise origin, grow in disturbed or maritime habitats and are sometimes apparently recent introductions or escapes from cultivation.

Island endemism is also interesting. In the New World there are single endemics in the Galapagos Islands (*Ipomoea habeliana* Oliv.), Hawaii (*I. tuboides* O. Deg. & Ooststr.), St Eustatius (*I. sphenophylla* Urb.) and Puerto Rico (*I. steudelii* Millsp.). None of the other smaller islands has a single endemic species^1^. However, high levels of endemism are reported from larger islands, that is Jamaica (4 species), the island of Hispaniola (7 or 8 species) and Cuba (15 species) by our conservative estimates. The level of endemism in Cuba is remarkable and only rivalled by the large continental countries of Mexico, Bolivia and Brazil. Most, but not all, of the endemic species of Cuba, Hispaniola and Jamaica as well as several species found in other Caribbean islands belong to a single clade of *Ipomoea* characterised by having coriaceous, concave, equal or only slightly unequal sepals, referred to below as the “coriaceous sepal clade”. There is a clear radiation of this clade in the islands of this region, but it is represented by only a few species in continental Central America and Mexico. The clade also boasts a number of unusual features uncommonly or never found elsewhere in *Ipomoea*; several species are remarkable for developing leaves on brachyblasts (most obviously *I. tenuifolia* (Vahl) Urb., *I. steudelii* Millsp. and *I. eggersiana* Peter but also *I. microdonta* J. R. I. Wood & Scotland described as new below), all four, perhaps coincidentally, remarkable for their small leaves. Others show a remarkable plasticity in leaf form, most notably *I. microdactyla* Griseb. and *I. clausa* Rudolphi ex Ledeb., but this is also a feature of the Cuban endemic *I. alterniflora* Griseb. discussed below. Deeply lobed corollas occur in some species (especially in *I. repanda* Jacq.) while contrasting corolla shapes as a result of different pollination syndromes (*I. eggersiana* and *I. steudelii*) are also noteworthy. These unusual features sometimes aid species delimitation but in other cases lead to uncertainty, most notably in the case of *I. horsfalliae* Hook. discussed below.

We consider there are 50 species of *Ipomoea* definitely occurring in Cuba with another nine excluded for one reason or another. These are listed below in six informal categories:

***Introduced species*** present in cultivation, which may or may not have escaped (6): *Ipomoea batatas* (L.) Lam., *I. carnea* Jacq. subsp. *fistulosa* (Mart. ex Choisy) D. F. Austin, *I. horsfalliae* Hook., *I. purpurea* (L.) Roth, *I. tiliifolia* (Desr.) Roem. & Schult., *I. tricolor* Cav.

***Widespread***, ***often weedy, species*** found in both the neotropics and palaeotropics, which are well established and which in several cases may be native (probably introduced species are indicated by asterisks) (14): *Ipomoea alba* L., *I. aquatica Forssk., *I. asarifolia* (Desr.) Roem. & Schult., *I. cairica (L.) Sweet, *I. corymbosa* (L.) Roth ex Roem. & Schult., *I. fimbriosepala* Choisy, *I. hederacea Jacq., *I. hederifolia* L., *I. indica* (Burm.) Merr., *I. nil* (L.) Roth, *I. obscura (L.) Ker-Gawl. (including *I. ochracea (Lindl.) G. Don), *I. quamoclit L., *I. setifera* Poir., *I. triloba* L.

***Widespread but uncommon American species***, possibly native in Cuba or of ancient introduction (3): *Ipomoea longeramosa* Choisy, *I. subrevoluta* Choisy, *I. heptaphylla* Sweet

***Widespread seashore species*** (4): *Ipomoea imperati* (Vahl) Griseb., *I. pes-caprae* (L.) R. Br., *I. sagittata* Poir., *I. violacea* L.

***Caribbean species*** (8): *Ipomoea carolina* L. (Cuba, Bahamas, SE United States), *I. jalapa* (L.) Pursh (also Ecuador), *I. meyeri* (Spreng.) G. Don (Circum-Caribbean except United States, south to Peru), *I. microdactyla* Griseb. (Cuba, Bahamas, Florida), *I. racemosa* Poir. (Cuba & Hispaniola), *I. tenuissima* Choisy (Florida, Cuba, Hispaniola, Mona Island), *I. tiliacea* (Willd.) Choisy (Circum-Caribbean except United States but also extending along the east coast of South America — reports of its occurrence in the palaeotropics may be errors), *I. trifida* (Kunth) G. Don. (Mesoamerica, Cuba, Trinidad, Caribbean coast of South America)

***Endemic or near endemic species*** (15): *Ipomoea alterniflora* Griseb., *I. argentifolia* A. Rich., *I. balioclada* Urb., *I. calophylla* C. Wright ex Griseb., *I. clarensis* Alain, *I. cubensis* (House) Urb., *I. fuchsioides* Griseb., *I. hypargyreia* Griseb., *I. incerta* (Britton) Urb., *I. jalapoides* Griseb., *I. merremioides* Alain, *I. microdonta* J. R. I. Wood & Scotland, *I. montecristina* Hadač, *I. passifloroides* House (also on Cayman Islands), *I. praecox* C. Wright.

***Excluded species*** (i.e. species accepted by Acevedo-Rodríguez & Strong (2012), Austin & Huaman (1996) and/or Sauget & Liogier 1957): *Ipomoea arnoldsonii* Urb. (= *I. fuchsioides*), *I. beyeriana* Urb. (probably = *I. microdactyla*), *I. carnea* subsp. *carnea* fide Acevedo-Rodríguez & Strong 2012, no specimen seen), *I. cordatotriloba* Dennst. (fide Acevedo-Rodríguez & Strong 2012, no specimen seen but occurrence possible), *I. excisa* Urb. (=*I. alterniflora*), *I. falkioides* Griseb. (mysterious species known only from the type, possibly not *Ipomoea*), *I. lindmannii* Urb. (sterile, unmatched), *I. perichnoa* Urb. (=? *I. jalapa*, no certain flowering material), *I. robusta* Urb. (sterile, unmatched).

## Materials and Methods

This paper is based principally on the study of specimens held in three herbaria, two in Cuba (HAC and HAJB) and one in Sweden (S), where Ekman’s specimens are held. We have seen many of Wright’s collections at HAC and at K and we have studied the particularly rich holdings of Jamaican collections at BM. Specimens have been examined using binocular dissecting microscopes to observe floral and indumentum details. We have also made considerable use of digital images, principally of types which are available through JSTOR (www.jstor.org/) but have also received images of important individual specimens held at IJ and LE.

### note on specimen numbers

Most specimens at HAJB are without collection numbers and are stored under accession numbers. In order to indicate this, specimens, principally from HAJB but occasionally at HAC, are cited by the herbarium acronym followed by the accession number.

Numbers in square brackets after Wright’s original numbers are those by which Wright’s collections are cited by Sauvalle (1870).

## 1. *Ipomoea alterniflora & I. obtusata*

These two species were described by Grisebach (1866: 202) on the same page in the same publication and there has been uncertainty over the application of the names ever since. Urban added to the confusion by treating specimens of *Ipomoea alterniflora* as *I. cubensis* (House) Urb. (Urban 1925: 427 – 8). A single species with the following synonymy is here recognised:

**Ipomoea alterniflora** *Griseb.* (Grisebach 1866: 202). Type: Cuba occ., *C. Wright* s.n. (holotype GOET000347!; possible isotypes GH!, HAC ex Herb. Sauvalle 1635!, NY!).

*Ipomoea obtusata* Griseb. (Grisebach 1866: 202). Type: *C. Wright* 3092 (holotype GOET000343!; isotypes GH!, K!, YU).

*Ipomoea obtusata* var. *latifolia* Griseb. (Grisebach 1866: 202). Type: *C. Wright* 3099 (holotype GOET?, not seen; isotypes G!, GH!, K!, NY!).

*Ipomoea excisa* Urb. (Urban 1924: 246), **synon. nov.** Type: Cuba, Prov. Pinar del Río [Havana?], Sierra de Anafe, Loma San Gabriel, 21 March 1920, *E. L. Ekman* 10558 (holotype S07-4426!).

*Ipomoea cubensis* sensu Urban (1925).

As understood here *Ipomoea alterniflora* is a variable species characterised by its glabrous stem, leaves and corolla. The leaves are usually ovate, cordate and shortly acuminate to an obtuse apex but are sometimes lobed as in the possible isotypes in HAC and NY. The inflorescence consists of lax, few- to many-flowered cymes. The sepals are subequal to slightly unequal, ovate, coriaceous, concave and glabrous. The corolla appears to be always narrowly funnel-shaped with included anthers. The corolla colour in the holotype is whitish-green (“albida” according to Grisebach) and this is clearly the same in the GH and HAC isotypes but the NY isotype is more darkly coloured and could be red. The seeds are pilose on the margins with long whitish hairs.

The most variable aspects of this species lie in the leaf shape. In the type of *Ipomoea obtusata* the leaves are ovate-elliptic with a rounded to cuneate base and obtuse apex. In the type of *I. excisa* the leaves are ovate but the apex is retuse. Although the extreme forms are rather distinct, there are many specimens which connect these with more common forms typified by *Wright* 3099 and the type of *I. alterniflora*. It is worth noting that almost from the time of their original descriptions by Grisebach, Wright (in Sauvalle 1870: 45) regarded *I. obtusata* and its varieties as synonyms of *I. alterniflora* but this was ignored by later authors.

*Ipomoea alterniflora* is endemic to western Cuba from where all collections come. It appears to be a plant of forest relics.

### selected specimens examined. cuba

**Isla de Juventud (Pinos):** *E. L. Ekman* 12563 (S); *A. Álvarez et al.* (HAJB 455570) — good match with *Wright* 3092, (HAJB). **Pinar del Río**: Mantua, Camarones, cima de Los Cabreros. *A. Álvarez et al.* (HAJB51183); Baños San Vicente, *N. L. Britton et al.* 7481 (NY); El Sapapo, Pinar de Sabanalamar, *A. Areces et al.* 28396 (HAC); Cabo Corrientes, Jaimanilas, *R. A. Quintana et al.* (HAJB 34218) — a good match with *Wright* 3092; Guanahacabibes, *J. Bissé et al.* (HAC, HAJB33208); Pinares de Cajálbana, La Palma, *Bro. Alain & J. Acuña* 1167, 1168 (HAC); ibid., *Bro. Alain* 6568 (HAC); ibid., *J. Acuña* 9086 (HAC); 16420 (HAC); *M. Yero* 25715 (HAC); Pinar del Rangel; Mogote de la Bandera, *Roig* 8358 (HAC). **Havana**: Loma de la Pita, San Miguel de Casanova, *Bro. León* 8388 (HAC); *Bro. León* 11588 (NY). Sierra de Anafe, *P. Wilson* 11417 (NY); *E. Ekman* 11494 (S); *Roig & Acuña* 14050 (HAC); ibid., Tibisí, *Bro. Alain* 6583 (HAC); ibid., Caimito, *M. E. Duharte et al.* (HAJB 60300) — good match with *Ekman* 10558; ibid., *J. Bissé et al.* (HAJB51278) — good match with *Ekman* 10558; Tetas de Managua, *H. A. Van Hermann* 318 (HAC). **Matanzas**: San Miguel de los Baños, *J. Bissé & Rojas* (HAJB 4522) — red-flowered. **Santa Clara**: Sierra Alta de Agabama, *R. Berazaín et al.* (HAJB 58044).

Although Urban (1925: 427) treated *Wright* 3099, *Wright* 12563 and *Ekman* 13494 as *Ipomoea cubensis*, this species is quite distinct as is clearly seen in the image of the type available through JSTOR and in the illustration in the *Bull. Torrey Bot. Club* (House 1908a: plate 2, at end). These both show a plant with deeply laciniate leaves, a broadly funnel-shaped corolla and strongly exserted stamens. We have seen no material that matches this very convincingly but *H. Manitz* (HAJB51284) from “Soroa cerca del Orquideario”, Pinar del Río, in western Cuba could well be this species. However, a search needs to be made in the gorge of the Yamurí River above Matanzas where the type was collected to relocate authentic examples of this species. In passing it should be noted that it is just possible that *I. cubensis* represents an example of extreme variability in *I. alterniflora*, a feature of the “coriaceous sepal” clade discussed in the introduction above and under *I. horsfalliae* below.

At Stockholm (S) there is a set of interesting specimens from the early 19th century which match the type of *Ipomoea obtusata*; these come from the Swartz herbarium and at least one of these was apparently collected by Forrström. According to Álvarez Conde (1958), Swartz visited Cuba so he, rather than Forrström, may have made these collections.

## 2. *Ipomoea calophylla*

*Ipomoea calophylla* was described by Grisebach in 1866 based on *Wright* 3098 collected in Cuba some years earlier. House (1908b) claimed this name was illegitimate, being a later homonym of *I. calophylla* Fenzl (1844) and renamed it *I. lacteola* House, a name which has been used by all subsequent authors. However, when checking the publication by Fenzl it became clear that *I. calophylla* Fenzl is a *nomen nudum* as no description of any kind was provided. Grisebach’s original name should stand as follows:

**Ipomoea calophylla** *C. Wright ex Griseb*. (Grisebach 1866: 204). Type: Cuba, *C. Wright* 3098 [1651] (holotype GOET000348!; isotypes BM!, G, GH!, HAC!, K!, S!, US!, YU).

*Ipomoea lacteola* House (1908b: 229), nom. superfl. Type: based on *I. calophylla* C. Wright ex Griseb.

## 3. *Ipomoea praecox*

**Fig. 1:**
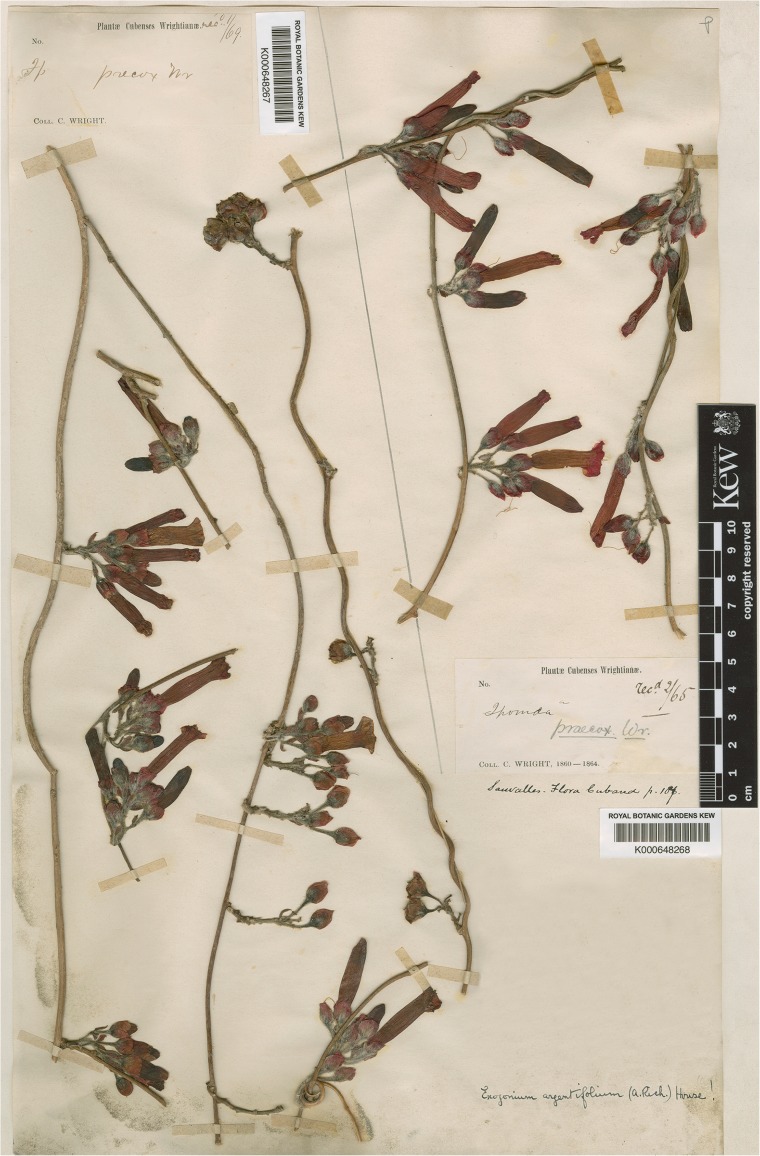
*Ipomoea praecox*. Image of specimen at Kew, provided by the Royal Botanic Gardens, showing distinctive leafless inflorescence, short cymes and reddish sepals, which are glabrous in the upper part.

**Fig. 2:**
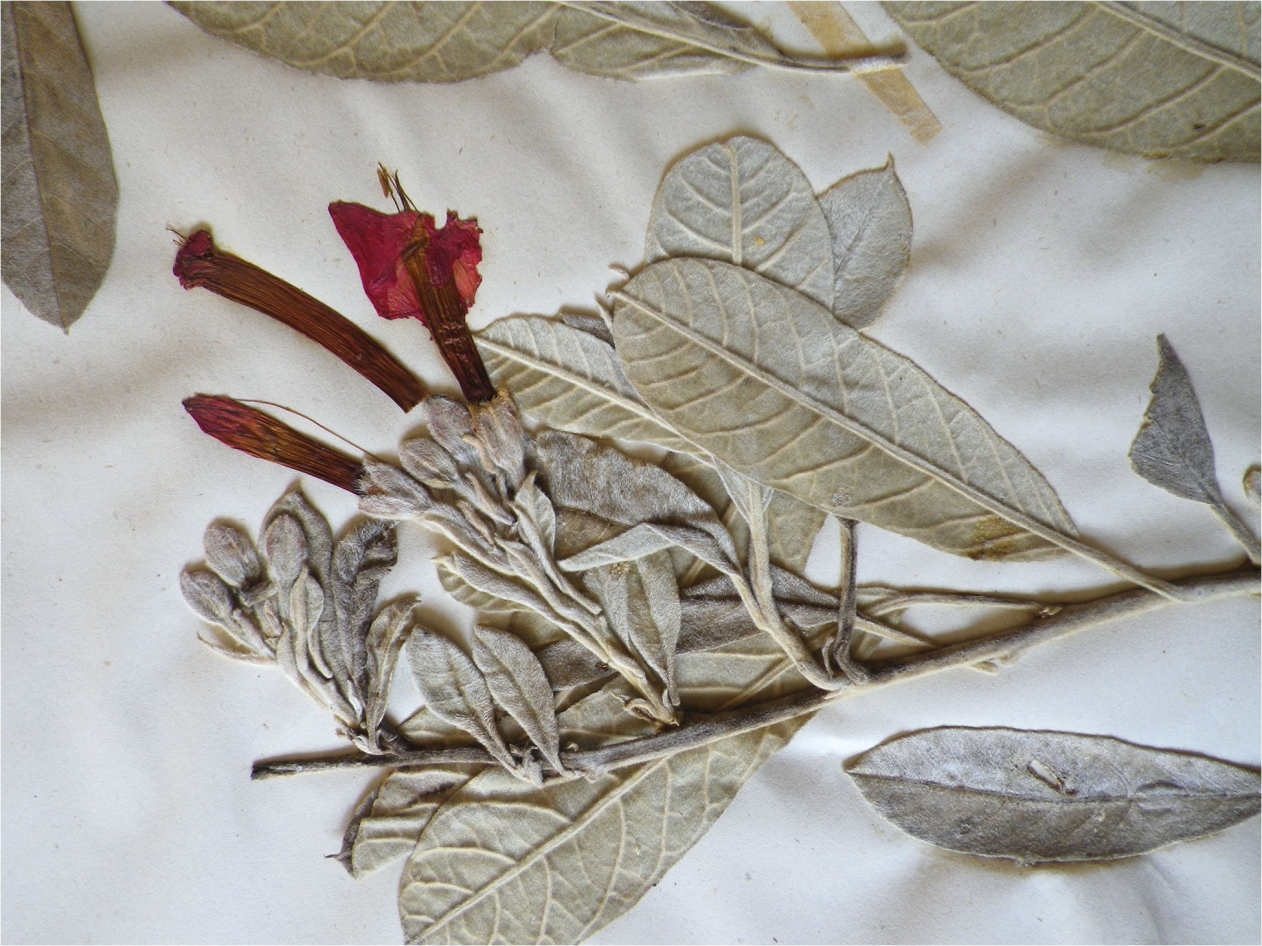
*Ipomoea argentifolia.* Photo of *Wright* 449 at Kew, showing leafy inflorescence with longer cymes and uniformly white, tomentose sepals.

Most of Charles (Carlos) Wright’s new species from Cuba were published by Grisebach (1866) in the *Catalogus plantarum cubensium*. Two species of *Ipomoea*, however, were described by Wright and published by Sauvalle (1870) in the *Anales de la Academia de Ciencias Medicas, Fisicas y Naturales de la Habana*. One of these was *Ipomoea praecox*, a species normally treated as a synonym of *I. argentifolia*. Examination of the type specimen and a series of collections in the Cuban herbaria shows that these two species are distinct. This was presumably also Wright’s opinion as he had already collected and recognised *I. argentifolia* (Sauvalle 1870). *I. praecox* differs from *I. argentifolia* in being leafless when flowering, in having the flowers arranged in dense, very shortly pedunculate, axillary subracemose cymes with up to six flowers, smaller, broadly ovate to suborbicular sepals 7 – 8 × 5 – 7 mm, which are hirsute only in the lower part and often reddish (Fig. 1). In contrast *I. argentifolia* has fully developed leaves at anthesis, the flowers arise on short, leafy axillary branchlets and the sepals are elliptic, longer, c. 10 – 11 × 6 – 7 mm, silvery in colour, the whole abaxial surface being covered in silvery hairs (Fig. 2). These differences are all clearly visible on the images of the types of the two species, which are available on JSTOR. Additionally the two species appear to be distributed differently. *I. argentifolia* is found on the Isla de Juventud (Pinos) off the west coast of Cuba but also occurs in the east of Cuba in the Sierra Maestra and Sierra de Nipe. In contrast, *I. praecox* is a plant of the Pinar del Río region.

**Ipomoea praecox** *C. Wright* in Sauvalle (1870: 46). Type: Cuba, [Pinar del Río], Lomas de Rangel, *C. Wright* 3646 [No. 1653 in Sauvalle 1870] (lectotype HAC!, said to have been collected on cliffs on hills at Balesteria in January (fide Wright in Sauvalle 1870: 46), designated here; probable isolectotypes GH!, K!, NY!).

### specimens examined. cuba

**Pinar del Río:** sin loc., *Mrs Earle* 4696 (HAC); Santa Cruz de los Pinos, *Bro. León* 22872 (HAC); ibid., *Bro. Alain* 466 (HAC, HAJB); La Palma, Loma Peluda de Cajalbana, *J. Bissé & H. Lippold* s.n. (HAC); Bahia Honda, Finca Toscano, *J. Bissé & H. Lippold* (HAJB18678); Candelaria, Sierra del Rosario, Loma Pelada de Cayajabos (del Mulo), *J. Bissé et al.* (HAJB48979); Las Villas, Soledad, *A. Gonzáles* 554 (BM).

## 4. *Ipomoea punctata*

*Ipomoea punctata* was a second species described by Wright (1870) after the publication of Grisebach’s *Catalogus Plantarum Cubensium* (1866). Although the epithet *punctata* had already been used by Poiret it was in fact quite appropriate for this species as the abaxial surface of the younger leaves is clearly punctate. Within the Cuban context, the species is very distinct, being annual, the corolla cream with a purple centre (hence Urban’s nomen novum *flavopurpurea*), the sepals with acute, strongly mucronate tips and the seeds shortly tomentellous and very different from the long pilose seeds of other Cuban endemics. We have never had material of this species available for sequencing so had not considered the possibility that it might be conspecific with a South American species until we noticed that *Ekman* 1876 (S) from Santa Clara in Cuba had been identified as *I. longeramosa* Choisy by O’Donell. Examination of Brazilian material of *I. longeramosa* showed that it matched the Cuban plant exactly and the leaves even have the same punctate lower surface which Wright observed. We have also been able to review an isotype of *I. dajabonensis* at Berlin through JSTOR and this is clearly also conspecific, something confirmed by the illustration in *La Flora Hispaniola* (Liogier 1994: 80). The full synonymy is set out below:

**Ipomoea longeramosa** *Choisy* (1845: 384). Type: Brazil, *Martius* s.n. (holotype M0185026; isotype M0185027).

*Ipomoea geranioides* Meisn. in Martius *et al.*, *Fl. Brasil.* 7: 276 (Meisner 1869). Type: Brazil, Mato Grosso, Cuiaba, *Riedel* 945 (lectotype NY00319188, designated here; isolectotype LE).

*Ipomoea punctata* C. Wright in Sauvalle (1870: 44 – 45), non *I. punctata* Pers. (Persoon 1805: 184). Type. Cuba, en las sabanas del potrero Manatí, Trinidad, *C. Wright* 3645 [1632] (lectotype K!, designated here; isolectotypes GH!, HAC!).

*Ipomoea flavopurpurea* Urb. (Urban 1903: 345), nom. nov. for *I. punctata* C. Wright, **synon. nov.**

*Ipomoea dajabonensis* Alain (Liogier 1978: 68), **synon. nov.** Type: Dominican Republic, en Manigua a la orilla de la carretera de Dajabón, *A. & P. Liogier* 27239 (isotype B).

### typification

*Martius* s.n. (M0185026) should be considered the holotype of *Ipomoea longeramosa* in preference to M0185027 as it bears Choisy’s annotation. There are two extant specimens (LE, NY) of *Riedel* 945 and we designate the NY specimen as lectotype of *I. geranioides* as it bears Meisner’s annotation “Ipomoea ?geranioides nob. (31./12./67)”. We have selected the Kew specimen of *Wright* 3645 as the lectotype of *I. punctata*. It could be argued that it is the holotype as what appear to be Wright’s notes describing the species are attached to the sheet.

### habitat and distribution

**Map 1:**
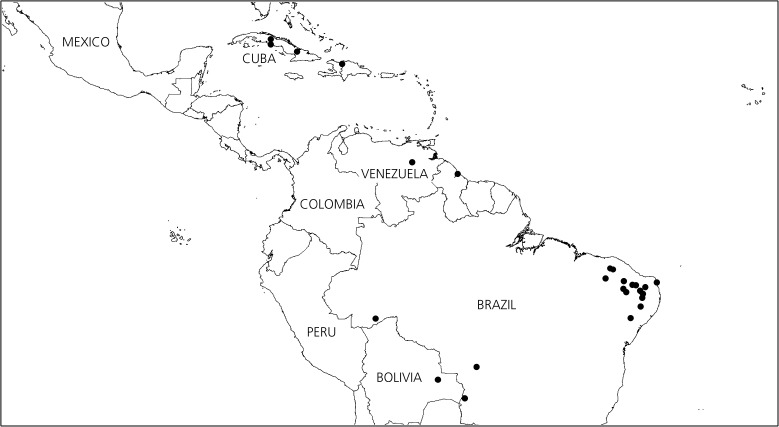
Distribution of *Ipomoea longeramosa* (•) in the Caribbean and South America, showing the disjunct distribution away from the core population in NE Brazil.

*Ipomoea longeramosa* is a somewhat weedy species growing in dispersed populations in semi-arid areas. It is most common in the arid NE of Brazil but occurs in scattered populations over a wide area of Brazil (Map 1). There are single collections from Bolivia (*Wood et al.* 24770 (K, LPB, USZ), Guyana (*Schomburgk* s.n. (K), Venezuela (*E. Holt & W. Gehringer* 156 (VEN)) and the Dominican Republic. In Cuba we are aware of five collections made in scattered localities over some 150 years. The possibilities of its occurrence elsewhere, particularly in the north of Colombia cannot be discounted.

The wide distribution but apparent rarity everywhere except in parts of NE Brazil is remarkable. This rather odd distribution pattern is also shared to some extent by two other species that occur in Cuba. One, *Ipomoea subrevoluta* Choisy, is a widespread but never very common South American species of stream sides in savanna which has been known in Cuba from the Isla de Pinos (Juventud) since Wright’s time. It occurs in scattered populations in Bolivia, Brazil, Colombia, Guyana, French Guiana, Paraguay, Peru, Trinidad and Venezuela, but is often only known from one or two locations in each country. The other is *Ipomoea heptaphylla* Sweet (= *I. wrightii* A. Gray). This is widespread in tropical and subtropical America from Argentina to the United States but likewise occurs in scattered populations and is generally uncommon.

### specimens examined. cuba

Sabanas del potrero Manatí, Trinidad, *C. Wright* 3645 (GH, HAC, K); Santa Clara, *E. Ekman* 18876 (S); [Granma] Aeropuerto Río Cauto, *Catasus* 2/95 (HAC40737); Victoria de Las Tunas 25 Nov. 1951, *J. Acuña & Montenegro* (HAJB17153, NY); Trinidad, carrera de Casilda a Playa Aneón, *J. Bissé et al.* (HAJB34707).

## 5. **Ipomoea montecristina**

Although this species was published nearly fifty years ago (Hadač 1970: 430), it has not yet been recognised in Cuba and was first picked up in the general literature by Acevedo-Rodríguez & Strong (2012). It represents a distinct species, which appears to be locally common in eastern Cuba.

**Ipomoea montecristina** *Hadač* (1970: 430). Type: Cuba, provincia Oriente, “montibus Montecristo dictis alt. circ. 800 m s. m., solo “laterit” dicto, legi 27.1.68”, *Hadač* 1279, (holotype PR, n.v.).

**Fig. 3:**
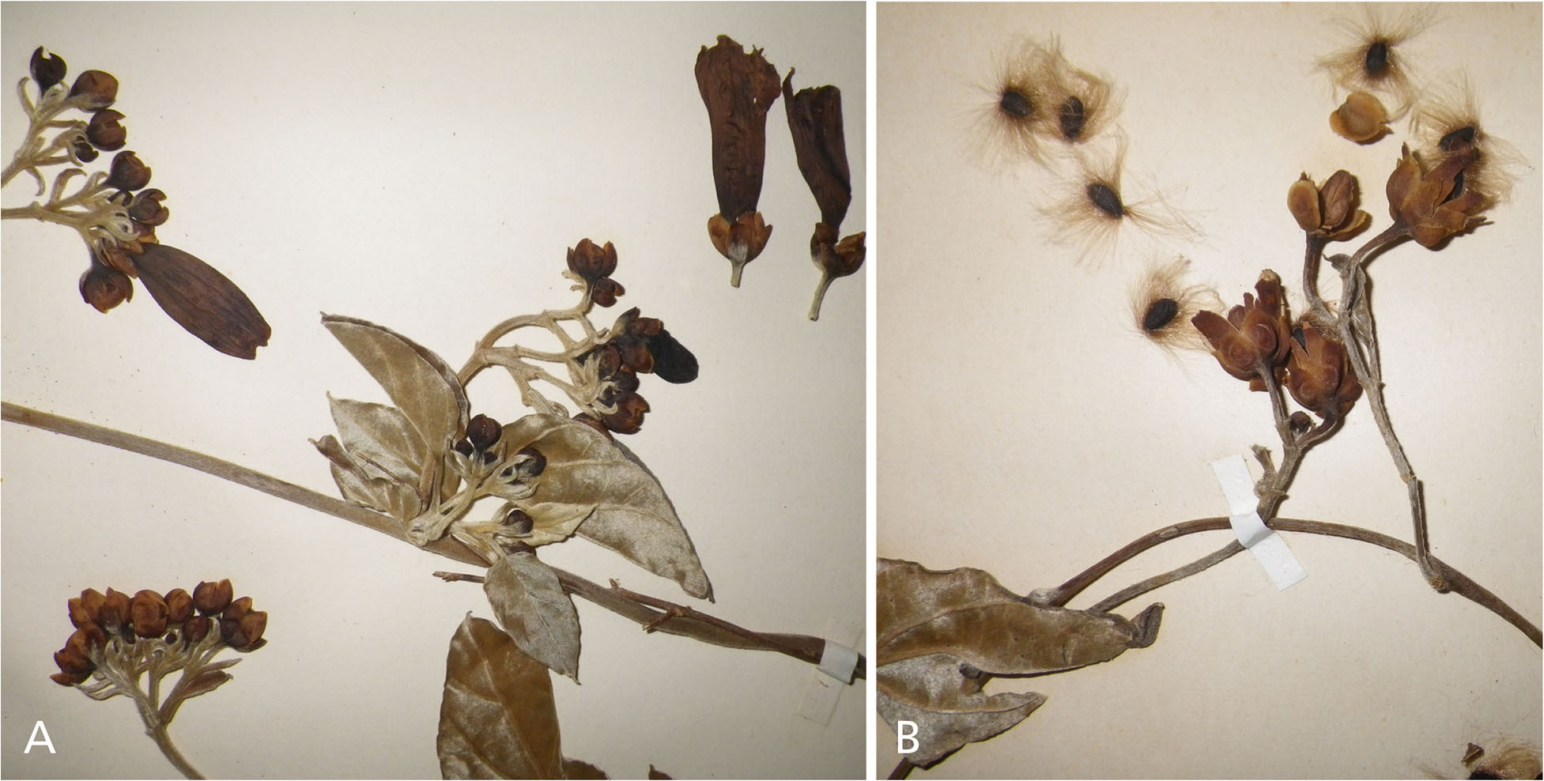
*Ipomoea montecristina*. **A** photo of inflorescence with leaves, sepals and corolla from *J. Bissé* HAJB20234; **B** photo of fruiting inflorescence showing seeds from *J. Bissé et al.* HAJB49387.

*Twining perennial*; *stems* sericeous, somewhat woody, and wiry. *Leaves* shortly petiolate, 2.3 – 6.5 × 0.8 – 3.2 cm, oblong-ovate, base cuneate, to weakly cordate, apex acute and shortly mucronate, adaxially dark green, densely pubescent, abaxially densely grey-velutinous, shiny; petioles 3 – 8 mm, sericeous. *Inflorescence* of pedunculate axillary cymes with up to 12 flowers; peduncles 1.4 – 3 cm, grey-tomentose; bracteoles linear, 3 – 6 × 1 mm, densely tomentose; secondary peduncles 3 – 12 mm, tomentose; pedicels 4 – 7 mm, thickened upwards and becoming less tomentose; sepals subequal, outer 5 – 6 × 3 – 4 mm, pubescent towards base, glabrescent, inner 6 – 8 × 4 mm, ovate, obtuse to rounded, reddish-brown, coriaceous, glabrous, margin narrow, pale. *Corolla* 3 – 3.5 cm, pink, glabrous, narrowly funnel-shaped; limb c. 1.5 cm diam.; stamens included, slightly unequal; anthers 2.5 mm. *Capsule* 10 – 11 × 5 – 6 mm, ovoid, glabrous, muticous; seeds 5 × 3 mm, blackish, long marginal hairs to 10 mm. Fig. 3.

### habitat & distribution

Apparently restricted to limestone outcrops, principally on Monte Cristi and near San Antonio del Sur in the Guantánamo Region.

### specimens examined. cuba

**Guantánamo Region: Monte Cristi,** 700 m, May 1968, *J. Bissé & E. Köhler* (HAJB8900); Monte Cristi altiplano, 700 m, May 1968, *J. Bissé & E. Köhler* (HAJB9335A); Pinar de Monte Cristi, sobre caliza, 500 – 600 m, 29 Oct. 1968, *J. Bissé & H. Lippold* (HAJB10218); ibid., 23 Aug. 1971, *J. Bissé* (HAJB20239); Felicidad de Yateras, pinar de la zona de Monte Cristi, sobre caliza, 23 Aug. 1971, *J. Bissé* (HAJB20234); ibid., 700 m, 13 May 1983, *J. Bissé et al.* (HAJB49387); ibid., Pinar de los Hondones, caliza, 850 m, 15 May 1980, *J. Bissé & A. Álvarez* (HAJB43272); Jamaica, pinar sobre calizas, Monte Cristi, 700 – 800 m, 10 Feb. 1979 (fr.), *J. Bissé et al.* (HAJB39180). **San Antonio**, Lomas al norte de San Antonio del Sur, 20 Aug. 1971, *J. Bissé* (HAJB20072); Lomas al este del Abra de Mariana, 6 km NE de San Antonio, 11 May 1980, *A. Álvarez et al.* HAJB 43089; ibid*., J. Bissé et al.* (HAJB48105); ibid. *J. Bissé et al.* (HAJB 36568); San Antonio del Sur, 4 km O – NO del pueblo Guantánamo, *J. Bissé et al.* (HAJB29883, HAC); Abra de Mariana, San Antonio del Sur, 9 Feb. 1979 *J. Bissé et al.* (HAJB39095); Reserva de Cupeyales, June 1965 (fl), *R. Alonso Olive* 25840 (HAC); región de Pinares de Moa, Baracoa, *Bro. León* 21291 (HAC); subida hacia la zona de Monte Libano, 300 – 500 m, May 1968, *J. Bissé & E. Köhler* (HAJB7924).

### note

This species is clearly part of the coriaceous sepal clade and has the distinctive sepals, glabrous corolla and pilose seeds typical of the clade. The corolla appears to be similar in shape to that of *Ipomoea alterniflora* but is always red.

## 6. A new species of *Ipomoea* endemic to Cuba

**Ipomoea microdonta** *J. R. I. Wood & Scotland*, **sp. nov.** Type: Camagüey, 2 – 7 April 1912, *N. L. Britton, E. G. Britton & J. F. Cowell* 13178 (holotype NY; isotypes MO, US).

http://www.ipni.org/urn:lsid:ipni.org:names:77165170-1

*Ipomoea cavanillesii* sensu Sauvalle (1870) and Sauget & Liogier [Bro. León & Bro. Alain] (1957).

**Fig. 4:**
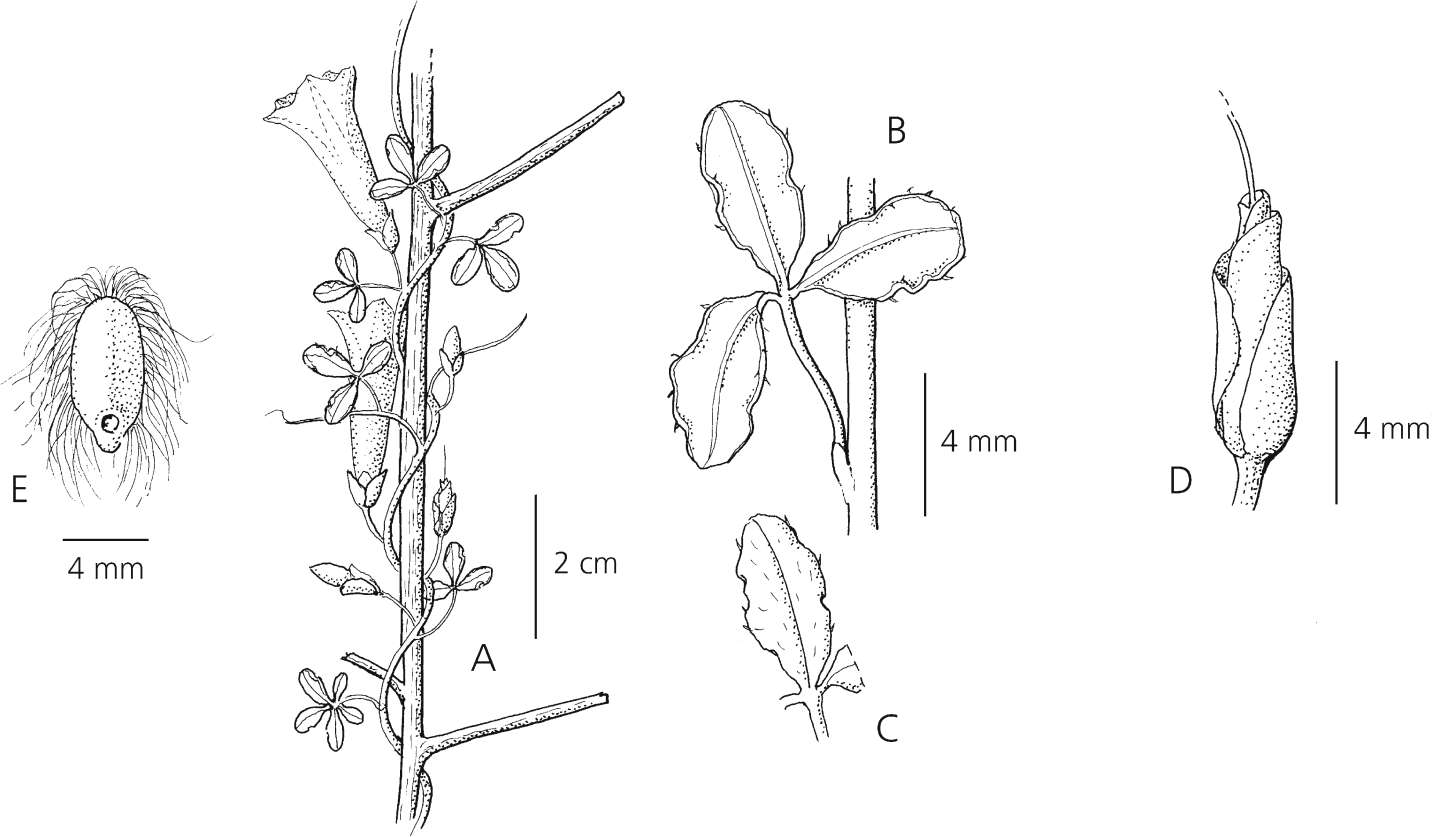
*Ipomoea microdonta*. **A** habit; **B** leaf; **C** terminal leaflet; **D** calyx; **E** seed. From *N. L. Britton et al.* 13178. drawn by rosemary wise.

Slender *twining perennial*; *stems* thin, wiry, woody, minutely asperous. *Leaves* petiolate, borne on small brachyblasts, 3-foliate, leaflets 3 – 10 × 1 – 5 mm, obovate-oblanceolate, apex obtuse to retuse, base cuneate, margin undulate, adaxially thinly hirsute, abaxially glabrous; petioles 2 – 8 mm. *Inflorescence* of axillary, pedunculate cymes with 1 – 4 flowers; peduncles short, 1 – 2 mm, bracteoles c. 2 × 0.75 mm, oblong, obtuse, caducous; pedicels 4 – 6 mm, glabrous; sepals unequal, glabrous, margins scarious, outer sepals 4 – 5 × 3 mm, elliptic-obovate, obtuse to rounded, inner sepals 6 – 7 × 4 mm, elliptic, rounded; corolla pink, subcylindrical to narrowly funnel-shaped, glabrous, 2 – 3 cm long; limb 1 – 1.5 cm diam., distinctly lobed; stamens and style included. *Capsule* c. 9 × 6 mm, ovoid, rostrate, glabrous; seeds 4 – 7 × 2.5 – 3 mm, blackish, glabrous but with dense long marginal hairs 5 – 10 mm in length. Fig. 4.

### recognition

Although originally identified as *Ipomoea cavanillesii* Roem. & Schult., a synonym of *I. cairica* (L.) Sweet, this species differs in many significant ways including the absence of stipule-like outgrowths at the base of the petiole, the tiny, minutely toothed leaves borne on short brachyblasts, the small corolla and the seeds with long marginal hairs. These characters, combined with the subequal, glabrous, rather rigid sepals, suggest it belongs to the “coriaceous sepal” clade. The minute dimensions of the palmately-divided leaves with small separate leaflets separate it from all other species with palmately divided leaves in this clade such as *I. carolina* and *I. microdactyla*.

### habitat & **distribution**

Endemic to the area around Camagüey in Cuba. Trailing and climbing on sandy plain.

### specimens examined. cuba

 sin. loc., *C. Wright* 3086 [1638] (K). Camagüey, 2 – 7 April 1912, *N. L. Britton, E. G. Britton & J. F. Cowell* 13178 (MO, NY, US); La Ciega, Caobillas, Camagüey, 25 June 1927, *J. Acuña* 1540 (HAC); Sabana de Croms, Camagüey, 6 April 1940, *Bro. León & M. Victorin* 17641 (HAC); Guaímaro, al norte de Monte Grande, Camagüey, 12 May 1976, *R. Berzaín, J. Bissé, M. Díaz, L. González & H. Lippold* (HAJB31501).

### conservation status

This species is seldom collected and appears to be limited to the Camagüey area but in the nearly complete absence of information about its frequency, or continued presence it can only be treated as DD (Data deficient) within IUCN (2012) guidelines.

### etymology

The epithet *microdonta* refers to the tiny marginal teeth of the leaves.

### note

Although we have not sequenced any example of this species, it almost certainly belongs to the “coriaceous sepal clade” based on its sepal, floral and seed morphology together with species such as *Ipomoea alterniflora, I. montecristina, I. balioclada* and *I. clausa.* The small leaves borne on brachyblasts suggest a close relationship with *I. tenuifolia*, *I. eggersiana* and *I. steudellii* the four species having distinct and complementary distributions in the Caribbean region.

## 7. Urban’s Cuban Ipomoeas

Urban (1924, 1925) described a series of new species of *Ipomoea* based on collections by Eric (E. L.) Ekman from Cuba. Most of these have never been collected again and remain problematic despite being included in the standard lists of Cuban endemics as good species (Sauget (Bro. León) & Liogier (Bro. Alain) 1957; Austin & Huaman 1996; Acevedo-Rodríguez & Strong 2012). One of us (JRIW) has seen all these types, which are housed in the Rijksmuseet in Stockholm (S), and also looked for matching material in the main Cuban herbaria (HAC, HAJB). We have evaluated these species as follows:

*Ipomoea arnoldsonii* Urb. (Urban 1925: 424)

This species is only known from the type collection (*Ekman* 18029) from Pinar del Río. It is a good flowering specimen and it seemed strange that it had not been recollected as it comes from the well-known area of Viñales in Pinar del Río. Examination of the type and the sequence of Ekman’s collections at Stockholm shows that *Ipomoea arnoldsonii* is a nearly glabrous form of *I. fuchsioides* with only a few hairs on the young stems and leaves. It was collected along with *Ekman* 18031 which is perfectly typical *I. fuchsioides* and shares exactly the same collection details. It seems that Ekman must have selected a nearly glabrous plant from a colony of *I. fuchsioides* and collected it under a separate number. We, therefore, regard it as a synonym of *I. fuchsioides*.

*Ipomoea balioclada* Urb. (Urban 1924: 245)

This is still only known from the type (*Ekman* 8080) and two further collections, *Ekman* 8749 and 14805, the latter the only collection with flowers. No recent collections of this species were found in either HAC or HAJB. Despite this and O’Donell’s tentative determination of it as *Ipomoea alterniflora* we accept *I. balioclada* as a good species. It is superficially identical to red-flowered forms of *I. alterniflora* but differs in the presence of conspicuous black glands on the stems. Geographically it is a species of extreme eastern Cuba from the Sierra Maestra above Daiquirí, 14 km E of Santiago de Cuba, a long distance from the known populations of *Ipomoea alterniflora*.

*Ipomoea beyeriana* Urb. (Urban 1924: 425)

This species is only known from the type (*Ekman* 18234) which is in fruit. O’Donell annotated the specimen with doubt as *Ipomoea alterniflora*. We think it is much more likely to be an entire-leaved form of *I. microdactyla* Griseb., based on the leaf shape and the reddish sepals. Urban (1925: 425) suggested an affinity with *I. fuchsioides* and it is not unlike *I. fuchsioides* var. *glabrata* Griseb., which we regard as a form of *I. microdactyla*. It is an even better match for *Wright* 3102, the type of *Exogonium microdactylum* var. *integrifolium* House, which we also regard as a synonym of *I. microdactyla*. *I. beyeriana* was collected in Pinar del Río where *I. microdactyla* is not uncommon.

*Ipomoea erosa* Urb. (Urban 1925: 425).

This is only known from the type specimen collected in the Sierra de Nipe in the extreme east of Cuba. We recognise this as a distinct endemic species distinguished by the greenish-white corolla and hirsute, mostly denticulate leaves. It should be relatively easily recognisable like *Ipomoea balioclada* even when sterile because of the distinctive leaves. No further specimen, however, has been seen despite search in the Cuban herbaria and collections of other species from the Sierra de Nipe.

*Ipomoea excisa* Urb. (Urban 1924: 246)

We consider this to be a not very distinct form of *Ipomoea alterniflora* with retuse leaves and have treated it as a synonym of that species above.

*Ipomoea lindmannii* Urb. (Urban 1924: 248).

This sterile shoot (*Ekman* 7508) was collected at Mir in eastern Cuba. No flowering or fruiting material has ever been found and it cannot even be certain that it is a species of *Ipomoea*. It is remarkable, however, for the shiny, silvery abaxial surface of the ovate-deltoid, cordate leaves. At HAC there is a similar sterile specimen (*Alberto Alonso* 26585) but this comes from Guanahacabibes, Pinar del Río, far from the type locality of *I. lindmannii.* Until flowering material can be found to confirm the identity of these plants, *I. lindmannii* should be treated as a dubious species of *Ipomoea*.

*Ipomoea perichnoa* Urb. (Urban 1925: 426)

Collected from the Guanahacabibes peninsula in Pinar del Río, the type of this species (*Ekman* 18781) is a fruiting specimen with densely lanate seeds, the hairs covering the whole surface of the seeds. Apart from the unusual seeds there is nothing in the type specimen to suggest it is distinct from *Ipomoea jalapa* (L.) Pursh, the species with which Ekman compared it (under the name *I. calantha* Griseb.). Two flowering specimens (*Ekman* 13521 and 18176) from different localities in Pinar del Río and Havana were cited as probably conspecific but these cannot be distinguished from *I. jalapa*. We have seen hardly any specimens of *I. jalapa* with seeds so it is very difficult to be certain that the seeds of *Ekman* 18781 lie outside the range of variation found in *I. jalapa*. They are similar to the seeds of the closely related *I. macrorhiza* Michx., which is endemic to the United States but unknown from Cuba so it seems quite likely that similar seeds are found in *I. jalapa*. *I. perichnoa*, therefore, remains an uncertain species although it is probably a form of *I. jalapa*, a species known from Pinar del Río and Habana in Cuba (Sauget & Liogier 1957: 240).

*Ipomoea robusta* Urb. (Urban 1925: 424).

This is based on a relatively unremarkable sterile shoot (*Ekman* 18220) collected in Pinar del Río. While this could be a species of *Ipomoea*, it too should be treated as a dubious species unless fertile matching material can be found.

## 8. Mrs Horsfall’s *Ipomoea*

Amongst the cultivated species of *Ipomoea* occurring in Cuba is *Ipomoea horsfalliae* Hook. This is grown in many tropical countries under various names including “*briggsii*” (after Sir Thomas Graham Briggs), “Lady Doorly’s Morning Glory”, “Cardinal Creeper” and “Prince Kuhio Vine” (Hawaii), the various names representing local names, cultivars or explanations of its supposed origins. It is a woody liana with digitate leaves composed of (3 –) 5 (– 7) sessile, glabrous leaflets and large branched inflorescences of very attractive pink flowers, viewable on numerous internet sites. The sepals are coriaceous, concave and glabrous, equal or somewhat unequal in size, thus placing *I. horsfalliae* in the “coriaceous sepal” clade with *I. mauritiana* Jacq. and many Caribbean species, something confirmed by our molecular studies.

**Fig. 5:**
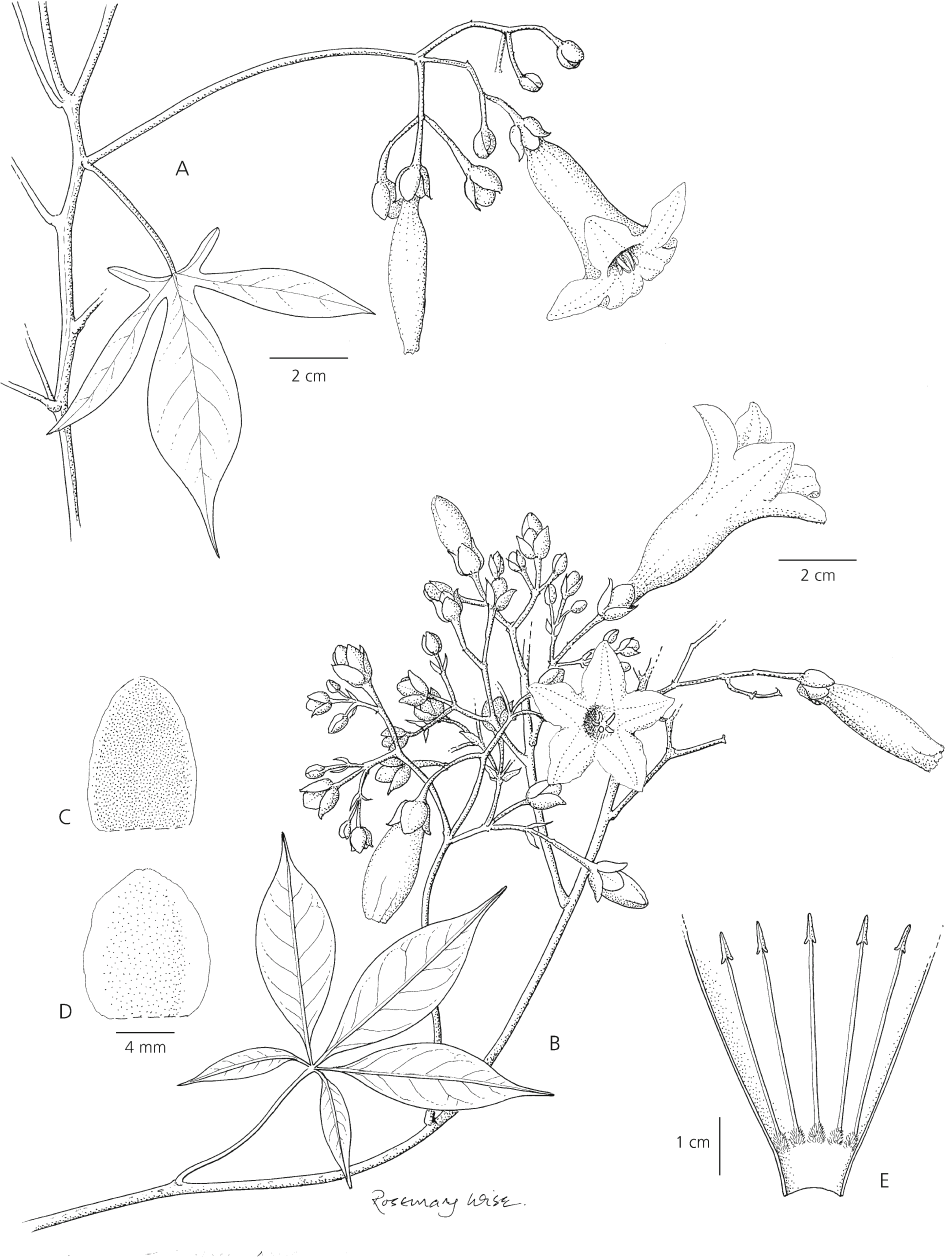
**A** cultivated plant of *Ipomoea horsfalliae* from Cuba, habit showing lobed leaves found in some cultivated forms. **B – E**. *Ipomoea lineolata* (form treated as *I. horsfalliae* in Jamaica); **B** habit; **C** outer sepal; **D** inner sepal; **E** corolla opened out to show stamens. **A** from *Jack* 4278; **B – E** from *Fosberg* 42712. drawn by rosemary wise.

*Ipomoea horsfalliae* shows many kinds of variation. The leaflets are usually sessile (as in the type) but in many forms the leaves are merely lobed (Fig. 5A). In some specimens the corolla is deeply lobed but in others it is much more shallowly lobed. This variation can be appreciated by viewing images on the internet. Powell (1979: 268) noted that one such specimen, *Fairchild* 2768 from St Lucia, appeared to be intermediate between *I. horsfalliae* and *I. repanda* and was presumably a hybrid between the two. There was “progression in leaf-lobing from 3 to weakly 7-lobed. Each flower had a slightly curved cylindrical corolla tube… with the limb ... lobed for its entire length, the pistil and stamens exserted.” She suggested that “Herbarium material often inaccurately (sic) labelled ‘*Ipomoea horsfalliae* (Cult)’ and collected throughout the tropics is mostly of similar hybrid material with lobed leaves and narrow-tubed flowers, scarcely to deeply lobed. The cultivated var. *briggsii* is perhaps this hybrid.” The existence of hybrids has also been mentioned by Acevedo-Rodríguez (2005: 167). The apparent rarity of fruits in cultivated plants together with extreme variation in corolla characters tends to support the view that cultivated *I. horsfalliae* is of hybrid origin. It seems that propagation is mostly from cuttings and very rarely from seeds.

The origin of *Ipomoea horsfalliae* has been uncertain from the beginning. Hooker (1834) reported that the seeds were thought to come “from either Africa or from the East Indies” and as recently as 2012, Acevedo-Rodríguez & Strong (2012: 241) suggested it was South American. However, a Caribbean origin has usually been proposed and this is supported by its inclusion in a clade of mostly Caribbean species with coriaceous sepals. Urban (1903) suggested it might have originated in Puerto Rico, where it grows “in moist forests of the Cordillera Central and in the zone of mogotes” but was treated as “possibly exotic and naturalized” (Acevedo-Rodríguez 2005: 167). However, the only place where plants similar to *I. horsfalliae* occur as definitely native species is Jamaica (Adams 1972; Austin 1980). Examination of a good number of specimens of apparently naturally occurring plants from Jamaica shows that these consistently differ from *I. horsfalliae* collected elsewhere in their petiolate leaflets. We were unable to find any Jamaican specimen that matched the numerous cultivated examples of *I. horsfalliae* and we do not think they represent the same taxon, even though the cultivated *I. horsfalliae* may well have arisen as a result of hybridisation involving the Jamaican plant. There is a clear need for more intensive study making use of DNA sequencing to resolve this issue. In the meantime we treat the cultivated plant as a distinct taxon of unknown but probably hybrid origin, nowhere occurring certainly as a native species.

**Fig. 6:**
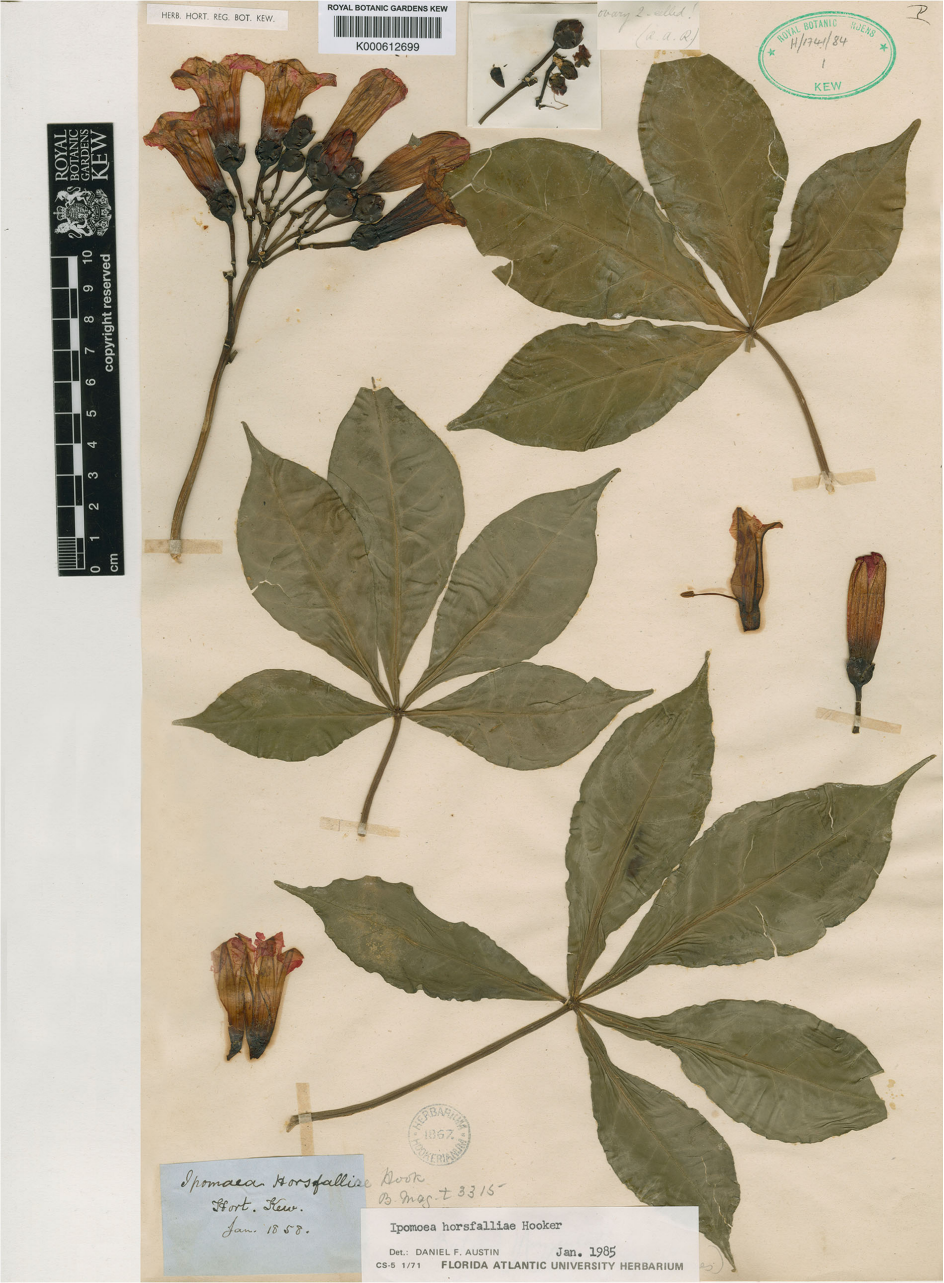
*Ipomoea horsfalliae*. Image of epitype at Kew, provided by the Royal Botanic Gardens.

**Ipomoea horsfalliae** *Hook*. (Hooker 1834: t. 3315). Type: Plate 3315 in *Bot. Mag*., lectotype designated here. Epitype: a cultivated plant from Hooker’s herbarium dated 1858 K000612699), designated here. Fig. 6.

*Convolvulus horsfalliae* (Hook.) D. Dietr. (Dietrich 1839: 664).

### habitat & distribution

Widely distributed throughout tropical and subtropical regions as a cultivated plant; apparently unknown in the wild but well naturalised in Puerto Rico (Acevedo-Rodríguez 2005).

### specimens examined. cuba

Santiago de Las Vegas, “June 1913” (sic), *H. A. van Hermann* 472 (HAC); Vedado, La Habana, Nov. 1915, *Bro. León* 8499 (NY); Harvard Tropical Garden, Soledad, Cienfuegos, Cuba, 12 March 1926, *J. G. Jack* 4278 (A); Central Moran, Camagüey, 16 Nov. 1930, *E. Vendrel Gili* 15544 (HAC). All the cited specimens have lobed leaves.

### typification

No type was preserved so the illustration by Mrs Horsfall in Curtis’ *Botanical Magazine* is here chosen as the lectotype. This image is slightly ambiguous about the fusion of leaflets at their base and is relatively few-flowered with inflorescences of 3 – 7 flowers. The exsertion of the stamens is slightly different in the two corollas, being distinctly exserted in one case but held at the mouth in the other. In order to clarify the characteristics of this species we have selected the specimen at Kew (K000612699) as an epitype (Fig. 6). This specimen originates from Hooker’s herbarium and is dated 1858, so, although it is not the original material used by Mrs Horsfall, it may well be derived from the same plant. In any case it matches the original painting well and no more suitable specimen is available.

### etymology

Unusually, *Ipomoea horsfalliae* was named for the artist, Mrs Horsfall, who prepared the painting from a plant cultivated by her husband’s “very skilful gardener Henry Evans at Everton…”

## A Jamaican complex

Although *Ipomoea horsfalliae*, as defined above, has not been recorded from Jamaica, the situation on the island is somewhat complicated by the presence of four closely related taxa, all clearly close to *I. horsfalliae*. Two of these have leaves divided into five leaflets, *I. horsfalliae* *sensu* Adams (1972) and *I. rubella* House, which Adams distinguished by differences in corolla size and the relative length of the sepals. Examination of a range of specimens show that these differences do not hold up and, as there exist intermediates, noted also by Adams, the two species cannot be separated satisfactorily. Two further species endemic to Jamaica differ only in having leaves divided into three leaflets. These are *I. lineolata* Urb. and *I. carmesina* Proctor. *I. lineolata* is reported to be relatively slender with few-flowered cymes but this is not apparent from herbarium specimens. *I. carmesina* is known only from the type and differs from *I. lineolata* in its many-flowered cymes, a characteristic placing it somewhat intermediate with the plant Adams called *I. horsfalliae*. None of the four species discussed here show any marked geographical or ecological pattern in their distribution in Jamaica and there seems no reason to treat them as separate species and so we treat them as single species under the oldest accepted name:

**Ipomoea lineolata** *Urb.* (Urban 1902 – 1903: 355). Type: Jamaica, *Wilson* “1126 aut 1155” (probably destroyed at B in 1943, no duplicate found at NY, neotype *G. R. Proctor* 10429 (BM001122860!), from Dolphin Head, Jamaica, designated here).

*Ipomoea grisebachii* Urb. (Urban 1903: 353), nom. illeg., non Prain (1894: 107). Type: Jamaica, Guy’s Hill, Moneague, *Alexander* s.n. (lectotype K000612811!, designated here).

*Ipomoea rubella* House (1907: 414). Type: based on *I. grisebachii* Urb.

*Ipomoea carmesina* Proctor (1982: 292). Type: Jamaica, [Trelawny], near Crown Lands road extension 4.5 – 5 miles NW of Troy, 7 Sept. 1974, *G. W. Proctor* 34169 (holotype IJ!).

### Notes

*Ipomoea lineolata* is quite variable particularly in the number of leaflets (3 – 7) and in their shape (lanceolate to oblanceolate, obovate or elliptic), but the leaflets are always narrowed to a petiole-like base (Fig. 5B). The corolla varies somewhat in length but is usually 5 – 6 cm long and the anthers are held at the mouth of the corolla and are not clearly exserted. The inflorescence is much branched in most plants with 5 leaflets (and also in the type of *I. carmesina*) but specimens with 3 – 5-flowered cymes are not uncommon. Some idea of the intermixture of the different leaf forms can be appreciated from the citations by parish of their occurrence in Jamaica, where they are most common in mountainous regions. It should be noted that all these wild populations have leaves divided into distinct leaflets with a petiole-like base including the oldest specimen of wild provenance we have seen (*Purdie* s.n.) collected in November 1843. Fig. 5B – E.

### selected specimens examined. jamaica

Clarendon, *G. L. Webster & G. R. Proctor* 5413 (BM — 3 leaflets), *F. R. Fosberg* 42712 (BM — 5 leaflets); Hanover, *G. R. Proctor* 10429 (BM — 3 leaflets); Manchester, *E. T. Robertson* 5552 (BM — 3 leaflets), *Purdie* s.n. (K — 5 leaflets); Portland, *H. A. Osmaston* 5101 (BM — 4 leaflets), *B. D. Morley & C. Whitefoord* 916 (BM — 7 leaflets, sterile); St Andrew, *G. R. Proctor* 18332 (BM — 3 leaflets), *T. G. Yuncker* 17184 (BM — 5 leaflets); St Ann, *A. Prior* s.n. [24/1/1850] (K — 3 leaflets), *G. L. Webster & G. R. Proctor* 5639 (A, BM, MICH — 5 leaflets); St Catherine, *G. R. Proctor* 32926 (BM — 3 leaflets), *G. R. Proctor* 34186 (BM — 5 leaflets); St James, *W. Stearn* 31 (BM — 5 leaflets); St Thomas, *C. D. Adams* 7262 (BM — 5 leaflets); Trelawny, *G. R. Proctor* 21374 (BM — 3 leaflets), *G. R. Proctor* 10599 (BM — 5 leaflets).

## 9. *Ipomoea saxicola*

*Ipomoea saxicola* Proctor was described in the same paper as *I. carmesina* (Proctor 1982). Examination of the isotype at BM shows that it resembles *I. ternata* Jacq. in all details except the densely hirsute stems and leaves. Other characters mentioned in the diagnosis are pink flowers (but “pink-tinged” flowers are found in *I. ternata* (Adams 1972: 610)) and the smaller seeds and capsules (but their dimensions overlap with those of *I. ternata*). We do not think that indumentum alone justifies recognition of *I. saxicola* as a distinct species (see Wood *et al.* (2015: 33) for the similar case of *I. subhirsuta* (Dammer) O’Donell), particularly as these populations occur only within the wider distribution of *I. ternata*. However, as the hirsute leaves are very distinct and do not seem to be bridged by intermediates with typical *I. ternata*, we are here recognising *I. saxicola* as a variety as follows:

**Ipomoea ternata** *Jacq*., (Jacquin 1797: 16) var. **saxicola** (*Proctor*), *J. R. I. Wood & Scotland*, **comb. et stat. nov.**

http://www.ipni.org/urn:lsid:ipni.org:names:77165177-1

*Ipomoea saxicola* Proctor, *J. Arnold Arbor*. 63: 292 (1982). Type: Jamaica, Clarendon Parish, Glenwood Springs, along road between Balcarres and Sunbury, 27 Sept. 1974, *G. R. Proctor* 34185 (holotype IJ, n.v.; isotypes BM!, GH!, NY!).

## 10. *Ipomoea cyanantha*

*Ipomoea cyanantha* Griseb. has long been recognised as a species endemic to Jamaica. Examination of collections shows clearly that this species is indistinguishable from *I. lindenii* M. Martens & Galeotti which is widespread on the American mainland. As in the case of *I. flavopurpurea*, this was noted long ago by O’Donell, who annotated *Harris* 12626 (NY) as *I. lindenii*, but this determination was ignored by all subsequent students of the genus. Formal synonymy and lectotypification are set out below:

**Ipomoea lindenii** *M. Martens & Galeotti* (1845: 264). Type: Mexico, [Veracruz], “dans les haies de la colonie de Zacuapan a 3000 pieds”, *Galeotti* 1360 (lectotype BR 00006973308, designated here; isolectotypes BR0000697264, G00227852, K000612747, P00625560).

*Ipomoea cyanantha* Griseb. (Grisebach 1864 [pub. 1862]: 469), **synon. nov.** Type: Jamaica, Mountains of St Andrews, *Purdie* s.n. (lectotype K00612707, designated here,).

### lectotypifications

Grisebach cited collections by Purdie and Wullschlägel after the description of *Ipomoea cyanantha*. We have designated the Purdie collection at Kew as lectotype as it is annotated by Grisebach and is the source of the quotation “fine blue” to describe the corolla colour quoted by Grisebach in the protologue and which was presumably the inspiration for the specific epithet “*cyanantha*”. We have also taken the opportunity to lectotypify *I. lindenii*. Martens & Galeotti cited two types, *Galeotti* 1360 and *Linden* 301 from Zacuapan in Mexico. We have selected *Galeotti* 1360 (BR00006973308) as lectotype as *Linden* 301 is not represented at Brussels and Galeotti is likely to have based the description on his own collection. There are isolectotypes at BR, G, K and P, the Kew specimen mounted on the lower half of a sheet on which *Linden* 301 is also mounted. The lectotype has been incorrectly annotated as the holotype.

As a result of the decisions in this paper the number of *Ipomoea* species endemic to Jamaica is reduced to four: *I. lineolata*, *I. ternata* Jacq., *I. jamaicensis* G. Don and *I. tenuifolia*.

## Footnotes

1 Although *Ipomoea walpersiana* Duchass. ex Urb. is treated as an accepted species endemic to Guadeloupe by Austin & Huáman (1996) we have been unable to trace a type and its identity is uncertain, even after a careful reading of the protologue.
